# The Impact of Pathological Margins on Basal Cell Carcinoma Recurrence: Does a Millimetre Matter?

**DOI:** 10.7759/cureus.82428

**Published:** 2025-04-17

**Authors:** Shahd Nour, Dalia Mohamed, Ouali Braham, Simon Wharton

**Affiliations:** 1 Plastic and Reconstructive Surgery, Dudley Group NHS Foundation Trust, Birmingham, GBR; 2 Burns and Plastic Surgery, University Hospitals Birmingham, Birmingham, GBR

**Keywords:** basal cell carcinoma, histopathological margins, margin width, recurrence, skin oncology

## Abstract

Background

Basal cell carcinoma (BCC) is the most common skin malignancy worldwide. Existing studies on histopathological margins and recurrence have lacked long-term follow-up and histopathological confirmation, leaving no clear consensus on the optimal margin to minimise recurrence, particularly in the UK population.

Objectives

This study aimed to evaluate the impact of histopathological margin width on BCC recurrence rates. Specifically, it sought to compare recurrence rates between lesions excised with ≤1 mm and >1 mm margins, assess recurrence patterns over a seven-year follow-up period, and determine whether wider margins had reduced the recurrence risk sufficiently to influence surgical guidelines and multidisciplinary team (MDT) discussions.

Methods

The Trust histopathology database was analysed to identify all patients with a histological diagnosis of BCC over a two-year period (January 2005 to December 2006). Histopathology reports for these patients were reviewed, and data on patient demographics, lesion site, BCC subtype, and histopathological margins were recorded, along with recurrence details over the subsequent seven years (January 2005 to December 2013).

Results

Of the 3,551 histopathology reports identified, complete records were available for 965 lesions. Pathological margins of ≤1 mm had been recorded in 269 lesions (narrow margin group), while 696 lesions had margins >1 mm (wide margin group). A total of 13 recurrences (4.8%) occurred in the narrow margin group compared to 14 recurrences (2.0%) in the wide margin group, showing a statistically significant difference (p = 0.017). The anatomical site of the BCC did not significantly impact recurrence, and BCC subtype was not a major determinant, with most subtypes showing no statistically significant association.

Conclusion

BCC recurrence can be influenced by several factors, including tumour location and size, histological subtype, patient immunosuppression, and follow-up limitations. Our findings confirm that achieving histopathological margins greater than 1 mm during BCC excision reduces the recurrence risk by more than half compared to excisions with ≤1 mm margins. Further research is needed to validate the factors contributing to recurrence, ensuring evidence-based surgical guidelines, MDT discussions, and optimal patient care for BCC management.

## Introduction

Basal cell carcinoma (BCC) is the most common skin malignancy worldwide, with a rising incidence in the Western world, particularly among men [1]. In the United Kingdom (UK), the age-standardised incidence rate (ASR) in 2010 was reported as 98.6 per 100,000 [2]. Later, Venables et al. [1] identified an increase in ASR to 269 and 411 per 100,000 in 2013 and 2015, respectively. These figures are likely underestimated, as some cancer information services freely acknowledge that they do not collect BCC data, while others collect only partial data. BCC recurrences can occur up to five years following primary excision [3], and the recurrence rates range from 0.8% to 10.1% [4,5]. Consequently, ensuring appropriate management of BCCs can lift a significant burden on healthcare resources.

Surgical excision remains the standard treatment to achieve histologically clear margins while minimising morbidity. The UK guidelines for BCC management currently recommend a 4 mm margin for low-risk BCCs and at least a 5 mm margin for high-risk BCCs [6]. Following excision, histopathological analysis determines the radial and deep excision margins, which are recorded in the histopathology report.

The British Association of Dermatologists (BAD) UK guidelines recommend that all adults with excised high-risk BCC and close histological margins (≤1 mm) be referred for multidisciplinary team (MDT) discussion to determine further management options, with patient preference playing a key role in decision-making [6]. Although previous studies have investigated histopathological margins and recurrence risk, follow-up was limited to one year, and there was no histopathological confirmation of recurrence [4]. Thus, there remains limited consensus on the optimal pathological margin required to prevent BCC recurrence, particularly within the UK population.

The aim of this study was to analyse a large cohort of patients who had undergone BCC excision. We identified those with histopathological confirmation of recurrence and compared recurrence rates between those with pathological margins of ≤1 mm and those with >1 mm, with a minimum follow-up of seven years.

## Materials and methods

A retrospective cohort study was conducted in the Plastic Surgery Department at Russell Hall Hospital to investigate recurrence rates in patients with histologically confirmed BCC over a seven-year follow-up period to capture late recurrences.

This study adhered to the Strengthening the Reporting of Observational Studies in Epidemiology (STROBE) guidelines for reporting observational studies. It was conducted as part of a quality improvement initiative in 2015 and represents one of the longest follow-up periods available for BCC recurrence.

Study population and data collection

The Trust’s pathology database was queried to identify patients diagnosed histologically with BCC over a two-year period (January 2005 to December 2006). A two-year recruitment period was chosen to ensure a sufficiently large cohort for meaningful comparison of recurrence rates between margin groups. All available histopathological reports related to BCC lesions were reviewed. Data containing demographics (gender and date of birth), date of primary excision, anatomical site of lesion, histopathological subtype, and both lateral and deep pathological margins were included.

In cases where the accuracy of the pathological margin on the primary report was uncertain (e.g., ≤1 mm, clear margin, widely excised, and narrow margin), cases were referred to a skin pathologist for further examination (n = 150) to ensure data accuracy.

Exclusion criteria

Patients with incomplete excision margins, incomplete datasets, lesions without subsequent excision where the latter lesion could not be confirmed as a recurrence, and duplicated records were excluded to maintain data accuracy and relevance.

Follow-up and recurrence data

Follow-up data on potential BCC recurrence were collected by reviewing the histopathological records of these patients from 1st January 2005 to 31st December 2013, to ensure a consistent seven-year follow-up for all cases. Recurrence was defined as histological confirmation of BCC at the same or an adjacent site (within 1 cm of the original excision site) within seven years of the primary excision.

Statistical analysis

The data were analysed, and p-values were calculated using SPSS software (IBM Corp., Armonk, NY). The primary objective was to determine whether narrow histopathological margins of 1 mm or less at the time of primary excision increased the risk of BCC recurrence. All results with p-values of <0.05 were considered significant in this study.

To compare recurrence rates between primary BCCs excised with wide margins versus those with narrow margins, a chi-squared test was conducted. Similarly, to assess the association between recurrence rates and other factors, such as gender distribution, anatomical site, BCC subtype, and timing of recurrence, individual chi-squared tests were performed. Pearson’s correlation test was used to identify any correlation between histopathological margin distance and time to recurrence. Surgeon variability was not considered a significant confounder, as the study focused on the outcome of the operation (histopathological margins) rather than the surgeon's technique.

To account for the confounding effects of anatomical site and BCC subtype, a multiple logistic regression analysis was used to allow for the adjustment of these variables while assessing the relationship between histopathological margins and BCC recurrence. The data were matched based on the available patient records and histopathological reports over the seven-year follow-up period, and the p-value was calculated to determine statistical significance. The model’s performance was assessed using McFadden’s R^2^ to determine overall fit.

Potential confounding factors such as tumour diameter and histopathologist interpretation were not accounted for in this analysis due to a lack of data availability.

## Results

A search of our Trust’s histopathology database identified 3,550 records of BCCs from January 2005 to December 2006. After application of the exclusion criteria, 965 records of completely excised BCCs from 750 patients remained. Of these, 269 lesions had a pathological margin of ≤1 mm at either the radial or deep margin, or both (narrow margin group), while 696 lesions had a pathological clearance of more than 1 mm at both margins (wide margin group). Of the 13 patients with narrow margin excisions and recurrence, 11 were male and two were female. Of the 14 patients with wide margin excisions and recurrence, eight were male and six were female, representing an overall male-to-female ratio of BCC recurrence of 2:1 (Table 1).

**Table 1:** Basal cell carcinoma recurrences: anatomical sites, histopathological margins and subtype, and time to recurrence.

	Age	Gender	Anatomical site		Radial margin (mm)	Deep margin (mm)	Histopathological subtype	Time to recurrence (years)
Wide margin group	72	M		Temple	4	4	Nodular	3
	71	M		Ear	3	2	Infiltrative	5
	79	F		Forehead	4	3	Nodular	1
	78	M		Post-auricular	6	5	Nodular	5
	53	M		Limb	1.5	2	Nodular	5
	70	M		Nose	6	4	Micronodular-superficial	7
	76	M		Scalp	5	5	Infiltrative	4
	76	F		Limb	8	4	Superficial	7
	76	M		Limb	9	3	Superficial	5
	58	F		Nose	2.5	6	Nodular	1
	73	F		Eyebrow	2	2.5	Infiltrative	6
	65	F		Temple	2.5	3	Nodular-cystic	1
	70	M		Post-auricular	2	1.5	Nodular	7
	65	F		Cheek	2.5	2	Nodular-superficial	5
Narrow margin group	56	M		Nose	0.5	1	Nodular-cystic	1
	78	M		Forehead	1	2.5	Nodular	6
	77	M		Eyelid	1	0.5	Nodular	4
	85	M		Trunk	1	≤1	Nodular	1
	58	M		Temple	2	1	Infiltrative-superficial	2
	77	M		Trunk	≤1	1	Infiltrative	3
	68	M		Pre-auricular	0.1	0.4	Nodular	2
	66	F		Nose	≤1	1	Nodular	4
	61	M		Temple	≤1	1	Superficial	7
	80	M		Nose	≤1	1	Nodular-infiltrative	3
	65	M		Forehead	1	1	Nodular-superficial	1
	72	M		Limb	0.7	1	Nodular-superficial	5
	73	F		Eyebrow	1	1	Nodular	3

Overall, 27 (2.8%) recurrences were recorded, with 13 (4.8%) recurrences in the narrow margin group and 14 (2.0%) in the wide margin group (Table 1). A chi-squared test comparing recurrence rates between margin types was significant (x2 = 5.678, p = 0.017).

Further analysis of histopathological margin distances in BCC excisions revealed a statistically significant, moderate positive correlation between radial and deep margin distances (r = 0.452, p < 0.001, 95% CI: 0.401, 0.501). This indicates that wider radial margins tend to be associated with wider deep margins, and narrower radial margins with narrower deep margins.

BCC recurrence can be influenced by factors such as tumour diameter, anatomical site, and histopathological subtype. Most recurrences occurred in the head and neck region, comprising 21 (77.8%) of all 27 recurrent BCCs. Within head and neck recurrences, 10 (76.9%) out of 13 recurrences were in the narrow margin cohort, and 11 (78.6%) out of 14 recurrences occurred in the wide margin cohort (Table 2). It is important to note that head and neck BCCs constituted 627 out of 965 primary BCCs excised (65.0%), and the results revealed no significant association between BCC recurrence and anatomical site (p = 0.547).

**Table 2:** BCC anatomical sites: recurrence rates and differences between narrow and wide margin groups. BCC: basal cell carcinoma.

Anatomical site	BCC total, n (%)	Wide margin group total, n	Wide margin recurrence, n (%)	Narrow margin group total, n	Narrow margin recurrence, n (%)	Total recurrence rate by site, n (%)
Head and neck	652 (67.6)	432	11 (78.5)	217	10 (76.9)	21.0 (3.2)
Cheek	87 (9.0)	63	1 (7.1)	24	0	1 (1.2)
Jaw	16 (1.7)	8	0	8	0	0
Ear	25 (2.6)	20	1 (7.1)	5	0	1 (4)
Eyebrow	13 (1.3)	8	1 (7.1)	5	1 (7.7)	2 (15.4)
Eyelid	72 (7.5)	31	0	41	1 (7.7)	1 (1.4)
Forehead	87 (9.0)	62	0	25	2 (15.4)	2 (2.3)
Temple	97 (10.1)	65	2 (14.3)	32	2 (15.4)	4 (4.1)
Lip	19 (2.0)	14	0	5	0	0
Neck	39 (4.0)	32	0	7	0	0
Nose	107 (11.1)	66	2 (14.3)	41	3 (23.1)	5 (4.7)
Post-auricular	38 (3.9)	22	2 (14.3)	16	0	2 (5.3)
Pre-auricular	29 (3.0)	22	0	7	1 (7.7)	1 (3.5)
Scalp	23 (2.4)	19	2 (14.3)	4	0	2 (8.7)
Trunk	168 (17.4)	145	0	23	2 (15.4)	2.0 (1.2)
Genital	2 (0.2)	2	0	0	0	0.0
Limb	143 (14.8)	117	3 (21.5)	26	1 (7.7)	4.0 (2.8)
Total	965	696	14 (2.0)	269	13 (4.8)	27.0

**Figure 1:**
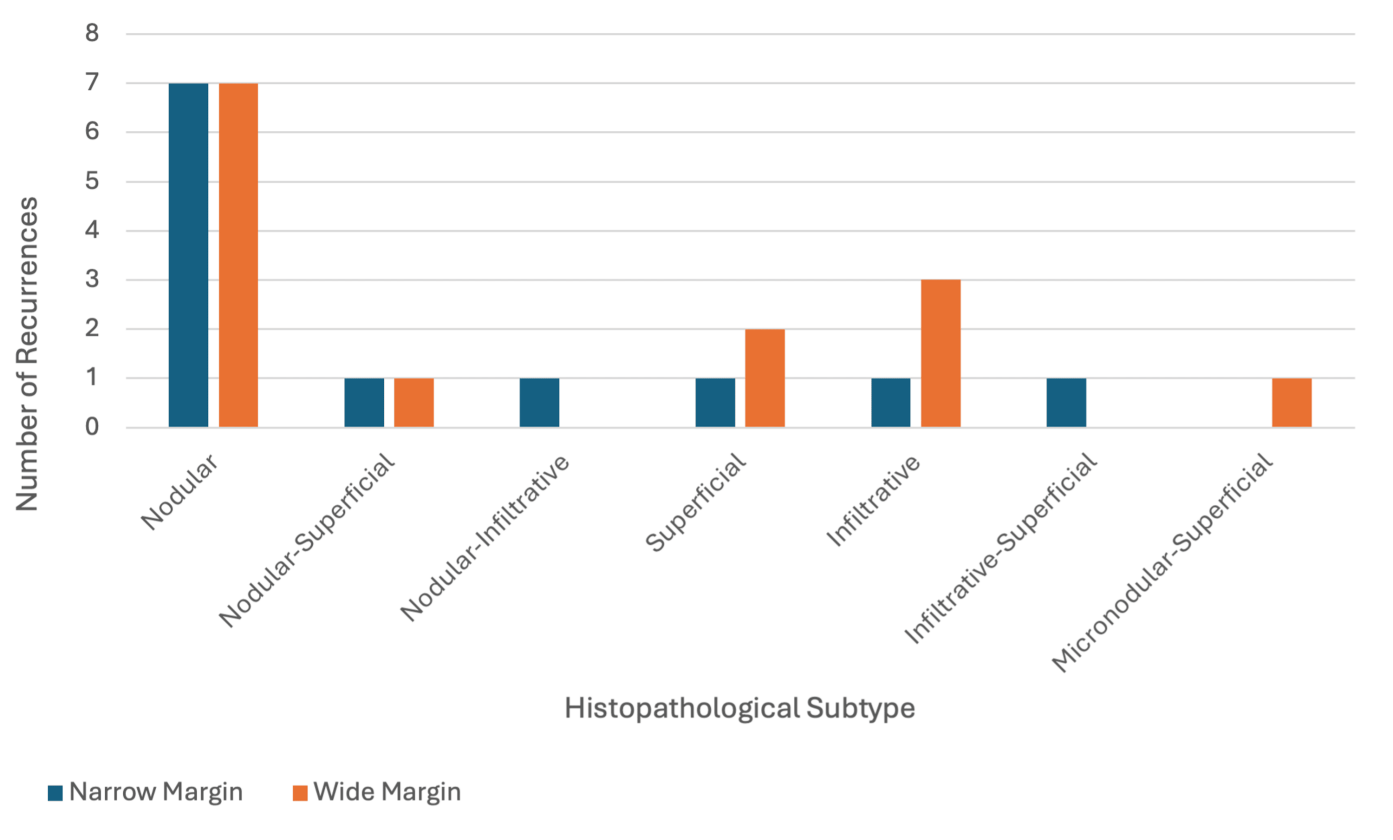
Comparison of recurrence risk in basal cell carcinoma histopathological subtypes between narrow and wide margin groups.

There was some variation in the recurrent BCC subtypes recorded (Table 1). Nodular BCC was the most prevalent subtype in both groups (Figure 1) and among all primary BCCs excised (n = 458, 47.5%) (Table 3). There was no significant association between the subtype of BCCs and the risk of recurrence (p = 0.980). This suggests that the subtypes of BCCs analysed did not significantly influence the likelihood of recurrence.

**Table 3:** Comparison of recurrence risk in basal cell carcinoma histopathological subtypes between narrow and wide margin groups.

Histopathological subtype	Total	Recurrent	Recurrence rate (%)
Adenoid	1	0	0
Basosquamous	8	0	0
Cystic	3	0	0
Fibroepithelial	2	0	0
Infiltrative	66	4 (6.06)	14.8
Keratotic	10	0	0
Micronodular	11	0	0
Morphoeic	17	0	0
Nodular	458	14 (3.06)	51.9
Pigmented nodular	6	0	0
Superficial	114	3 (2.63)	11.1
Ulcerative	12	0	0
Mixed histopathology:			
Infiltrative-keratotic	3	0	0
Infiltrative-morphoeic	8	0	0
Infiltrative-micronodular	2	0	0
Infiltrative-micronodular-superficial	3	0	0
Infiltrative-superficial	10	1	3.7
Infiltrative-ulcerative	24	0	0
Infiltrative-ulcerative-keratotic	2	0	0
Infiltrative-ulcerative-superficial	1	0	0
Morphoeic-keratotic	1	0	0
Morphoeic-superficial	1	0	0
Micronodular-keratotic	2	0	0
Micronodular-superficial	15	1	3.7
Nodular-adenoid-cystic	1	0	0
Nodular-basosquamous	2	0	0
Nodular-cystic	49	0	0
Nodular-cystic-superficial	1	0	0
Nodular-infiltrative	30	1	3.7
Nodular-infiltrative-keratotic	1	0	0
Nodular-infiltrative-superficial	6	0	0
Nodular-infiltrative-cystic	1	0	0
Nodular-keratotic	3	0	0
Nodular-morphoeic	4	0	0
Nodular-multinodular	2	0	0
Nodular-superficial	46	3	11.1
Nodular-ulcerative	19	0	0
Nodular-ulcerative-cystic	2	0	0
Nodular-ulcerative-keratotic	2	0	0
Nodular-ulcerative-superficial	1	0	0
Pigmented-adenoid-cystic	1	0	0
Ulcerative-keratotic	2	0	0
Ulcerative-morphoeic	2	0	0
Ulcerative-superficial	1	0	0
Adenoid-cystic	9	0	0
Infiltrative-keratotic	3	0	0
Infiltrative-morphoeic	8	0	0
Ulcerative-superficial	2	0	0
Total	965	27 (2.8%)	100%

**Figure 2:**
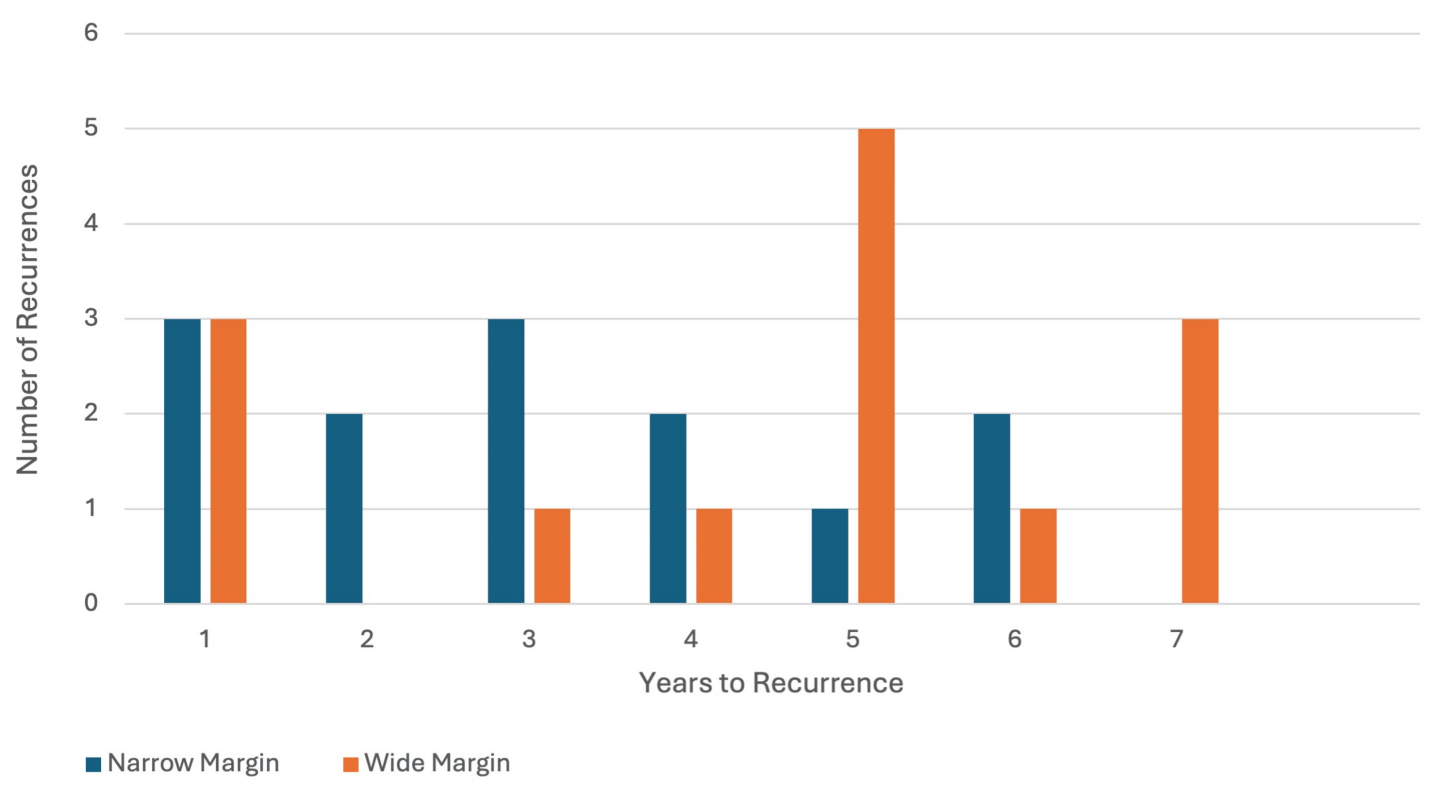
Comparison of recurrence delay between narrow and wide margin groups.

The time from excision to recurrence remained relatively consistent across both groups, ranging from one to seven years postoperatively (Figure 2). A mild positive correlation (r = 0.315) was observed, showing no distinct early vs. late pattern. However, this was not statistically significant (p = 0.109).

The age distribution between the recurrence groups showed some differences, with the narrow margin group ranging from 53 to 85 years and the wide margin group ranging from 58 to 81 years. This difference did not reach statistical significance (p = 0.078) with regard to recurrence.

We adjusted for potential confounders using multivariable logistic regression analysis, incorporating margin group, anatomical site, and BCC subtype as covariates to account for their influence on BCC recurrence. This showed that a narrow surgical margin (≤1 mm) is a significant risk factor for BCC recurrence compared to wide margin excision. Anatomical site did not significantly impact recurrence, and BCC subtype was not a major determinant, with most subtypes showing no statistically significant association. However, the model's overall fit was poor (McFadden R² = 0.052, p = 0.742), suggesting that additional factors, such as tumour size, patient immunosuppression, and histopathologist’s interpretation, not included in the model, may influence recurrence.

## Discussion

According to the National Comprehensive Cancer Network, risk factors associated with an increased risk of BCC recurrence include tumour localisation within the high-risk "H" zone (orbital, nasal, nasolabial fold, and auricular areas), larger tumour size, aggressive histological subtypes, and incomplete histopathological margins [3].

Regarding histopathological margins, current UK guidelines recommend that all adults with excised high-risk BCC and close histological margins (≤1 mm) should be referred for MDT discussion to determine further management options, incorporating patient preference into decision-making [6].

This guidance has been shaped by evolving evidence on the importance of histopathological margins, yet at the time of its release, there were limited data to support specific recommendations regarding optimal excision margins. A Cochrane review looking at cutaneous BCC interventions highlighted that recurrences can occur up to five years post excision, explaining the rationale for selecting a minimum seven-year follow-up in our study to capture late recurrence patterns in our cohort [7].

BCCs excised with a margin of ≤1 mm had a 2.4 times higher risk of recurrence compared to those excised with >1 mm margins, with a statistically significant p-value of 0.017. This finding indicates the sample was sufficiently powered and aligns with previous research correlating histopathological margins and recurrence. A three-year study of 539 lesions defined recurrence as lesions reappearing at the same site at least one year after surgery, albeit without histological confirmation. Despite this limitation, a greater than two-fold increase in recurrence risk was observed for BCCs excised with ≤1 mm margins compared to those with 1-3 mm margins [4]. Furthermore, no recurrences were reported in lesions excised with >3 mm margins, suggesting further benefits of achieving wider excision margins [4]. Similarly, a 1-mm cut-off value was identified by another study, below which the recurrence rate increased, although the extent of this increase was not quantified [8].

While recurrence rates in completely excised BCCs have been reported as high as 10.1% [5], our study demonstrated an overall recurrence rate of 2.8% (n = 27) recorded among excised primary lesions. Another study reported a recurrence rate of 0.8% (n = 72) [4]. This difference may have been due to variations in follow-up duration, population characteristics, or surgical techniques between studies.

Multiple studies have highlighted a higher recurrence risk in the head and neck region [4,5,8,9]. In our study, 21 (77.8%) recurrences occurred in the head and neck area, with a similar distribution between the narrow and wide margin groups. Another study also reported 68 (92%) recurrences occurring in this region, with further anatomical site-specific analysis [4].

Nodular BCC was the most commonly diagnosed recurrence type in both studies. In our cohort, nodular BCCs accounted for 50% (n = 14) of recurrences, while another study reported a 40% (n = 29) recurrence rate for completely excised nodular BCCs.

Time to recurrence varied across studies, but differences between margin groups were not statistically significant. In our study, 21 (82%) recurrences developed within five years, whereas Yildizdal et al. reported 66 (92%) recurrences occurring within the first five years following primary surgical excision [4]. An independent analysis identified a median time to recurrence of 3.5 years [7].

While patient age varied between the narrow and wide margin groups, this difference was not statistically significant, aligning with previous findings in the literature [4]. Radial and deep margins did not reliably predict each other, suggesting that achieving wide margins in one dimension does not necessarily ensure adequate clearance in the other.

These findings are particularly relevant within the broader context of rural plastic surgery, where access to specialist care, multidisciplinary teams, advanced histopathological analysis, and reliable follow-up is often limited. In the United States, an estimated 54 million people reside in designated health service areas with either no plastic surgeon or fewer than one plastic surgeon per 100,000 population. Consequently, higher recurrence rates associated with narrow excision margins risk placing additional strain on already overburdened rural services [10].

Given the significantly higher recurrence rate observed in our study, guidelines should consider the benefit of achieving wider excision margins to reduce the need for re-excisions and alleviate the healthcare burden. However, while wider margins lower recurrence risk, this must be balanced against morbidity, cosmetic concerns, and anatomical constraints. In cases where achieving a greater than 1 mm histopathological margin is impractical, such as on the scalp or nose, Mohs micrographic surgery or adjuvant therapies may be preferred.

Patient involvement in shared and informed decision-making is essential, offering the option to monitor rather than undergo immediate re-excision when narrow histopathological margins are identified following primary excision.

Limitations

The study had several limitations. Its retrospective design limited control over confounding factors. Although the multivariate logistic regression analysis adjusted for key factors influencing BCC recurrence, the overall model fit was poor, indicating limited predictive power. This suggests that other confounders, such as tumour size and perineural invasion, may influence recurrence but were not accounted for due to a lack of available data.

The study was subject to selection bias as patients treated with non-surgical methods were excluded. Patients lost to follow-up or those who presented elsewhere may have been missed, leading to an underestimation of recurrence rates. Variability in histopathological interpretation could have influenced margin assessment.

## Conclusions

This study, based on a large patient cohort with long-term follow-up, provides valuable insights into BCC recurrence following surgical excision. BCCs excised with a ≤1 mm margin had a 2.4 times higher recurrence risk than those with >1 mm margins, with no significant differences in anatomical site of BCC or BCC subtype between groups. These findings highlight the burden of higher recurrence rates, leading to more follow-up, re-excisions, and strain on healthcare resources, and support evidence-based margin guidance, particularly in settings with limited access to follow-up care. Achieving wider margins, where possible, may help reduce recurrence and ease pressure on skin cancer services.
